# Left supraclavicular parathyroid carcinoma: diagnostic difficulties through a case report

**DOI:** 10.11604/pamj.2024.48.106.41700

**Published:** 2024-07-15

**Authors:** Moad El Mekkaoui, Zainab Benyahia, Hafsa Elouazzani, Razika Bencheikh, Anas Benbouzid, Abdelilah Oujilal, Nadia Cherradi, Leila Essakalli

**Affiliations:** 1 Ear, Nose, and Throat, Head and Neck Surgery Department, Hospital of Specialties, Mohammed V University, Rabat, Morocco,; 2 Laboratory of Anatomical Pathology, Hospital of Specialties, Mohammed V University, Rabat, Morocco

**Keywords:** Parathyroid, carcinoma, surgery, case report

## Abstract

Parathyroid carcinoma is a very rare malignant tumour of the parathyroid gland, accounting for less than 0.005% of all cancers, and less than 1% of the aetiologies of primary hyperparathyroidism. This case report aims to describe the incidental discovery of a non-secreting parathyroid carcinoma in a left supraclavicular location and to report on the diagnostic difficulties encountered, together with a review of the literature and current management issues. A 43-year-old female presented with a left supraclavicular swelling with no associated signs. Imaging revealed left supra-clavicular adenopathy (Troisier lymph node) associated with a hetero-multi-nodular goiter classified EU-TIRADS 5. The abdominopelvic region was strictly normal. The patient underwent a total thyroidectomy with a left mediastino-recurrent curage and a homolateral laterocervical functional curage. Pathological examination was consistent with parathyroid carcinoma in the left supraclavicular region.

## Introduction

Parathyroid carcinoma is a very rare malignant tumour of the parathyroid gland, accounting for less than 0.005% of all cancers, and less than 1% of the etiologies of primary hyperparathyroidism [1,2]. This slowly progressing cancer poses a real diagnostic and therapeutic problem, given the lack of clinical and biological specificity enabling it to be easily differentiated from parathyroid adenoma. Curative treatment relies on surgery, and the role of adjuvant therapy is still a matter of controversy [3]. In this work, we present a case report of the incidental discovery of a non-secreting parathyroid carcinoma in a left supra-clavicular location and report on the diagnostic difficulties encountered, together with a review of the literature and current management issues.

## Patient and observation

**Patient information:** this is a 43-year-old patient, married with 3 children, housewife, of North African origin, epileptic for 16 years under treatment, with no other particular medical or surgical history, such as cervical irradiation or family history, who presented for one year with a left basicervical swelling progressively increasing in volume, with no signs of associated dysthyroidism or compression, nor rhinological, cardiac, digestive or urological signs, nor bone pain, all evolving in a context of preservation of general condition with no associated asthenia.

**Clinical findings:** clinical examination revealed a mass located in the left supraclavicular fossa, hard, stony, poorly limited, painless to palpation, fixed in relation to the superficial and deep planes, 4 cm in diameter, with no inflammatory signs opposite. Anterior rhinoscopy and nasal endoscopy were performed, and both were strictly normal. The rest of the ear nose and throat (ENT), abdominal and gynaecological examinations were unremarkable.

**Figure 1:**
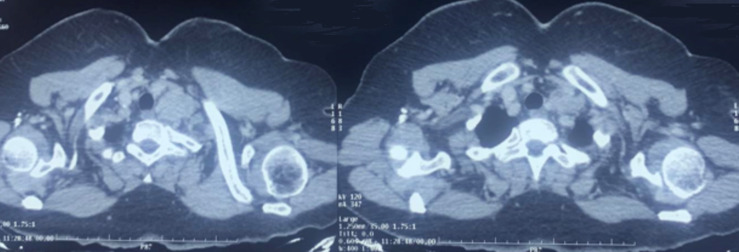
axial C+ cervical CT scan suggestive of left supraclavicular adenopathy

**Diagnostics assessment:** a radiological work-up was then ordered; cervical ultrasound revealed two poorly limited left supra-clavicular adenopathies with irregular contours, containing anechogenous areas associated with necrosis, without individualization of a central fatty hilum, forming a magma, richly vascularized in an anarchic manner on dopler, measuring 28X20mm and 21X32mm respectively, raising the suspicion of a trigeminal ganglion. Ultrasound also suggested a heteromulti-nodular goiter with two nodules: a left mediolobar nodule, strongly hypoechoic, peripherally vascularized, containing microcalcifications classified EU TIRADS V measuring 30X17mm and a right lower lobar nodule classified EU TIRADS III measuring 6X12mm. The radiological work-up was completed by a cervico-thoraco-abdomino-pelvic computed tomography (CT) scan to study the relationship of the adenopathies to adjacent structures (Figure 1), and to rule out a morphological abnormality at the abdomino-pelvic level. As for the biological work-up, a haemogram was requested, which came back without any abnormalities. An intra-dermo reaction to tuberculin was performed, showing an induration 12mm in diameter. The thyroid hormone profile was strictly normal, with TSHus at 0.764 mUI/L, T3 at 3 pg/ml and T4 at 0.94 ng/dl. The blood ionogram was without abnormalities, with corrected calcemia at the limit of normal (107mg/l).

**Figure 2:**
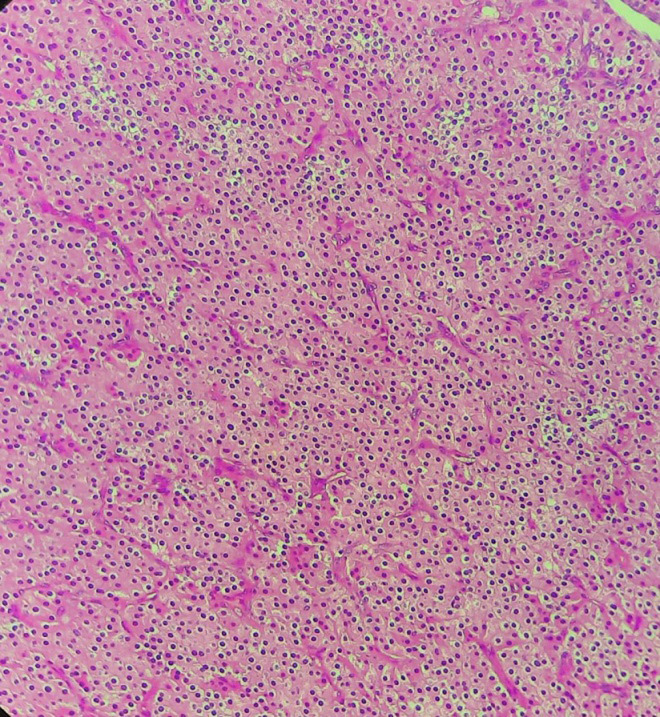
monomorphic tumor proliferation in a small vascular endocrine-like stroma (HEX10)

**Figure 3:**
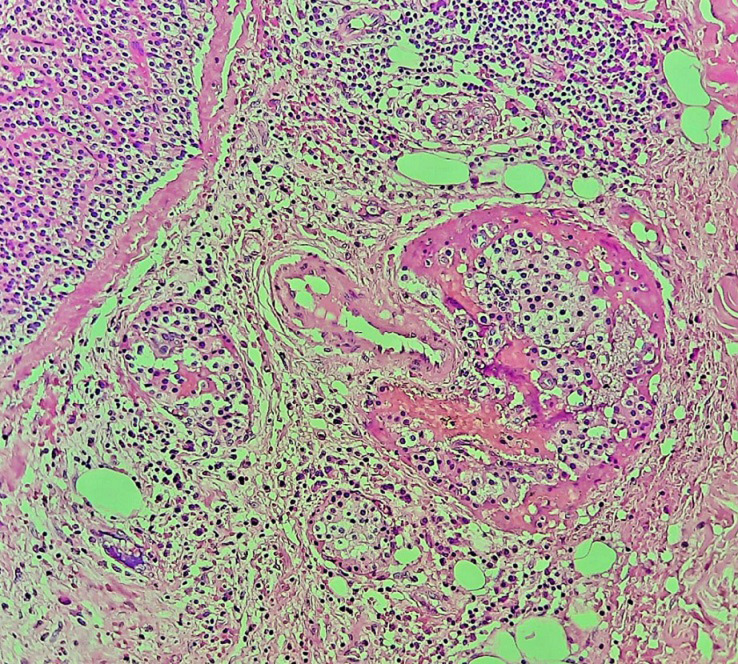
proliferation infiltrates adjacent connective tissue with angio-invasion (HEX20)

**Figure 4:**
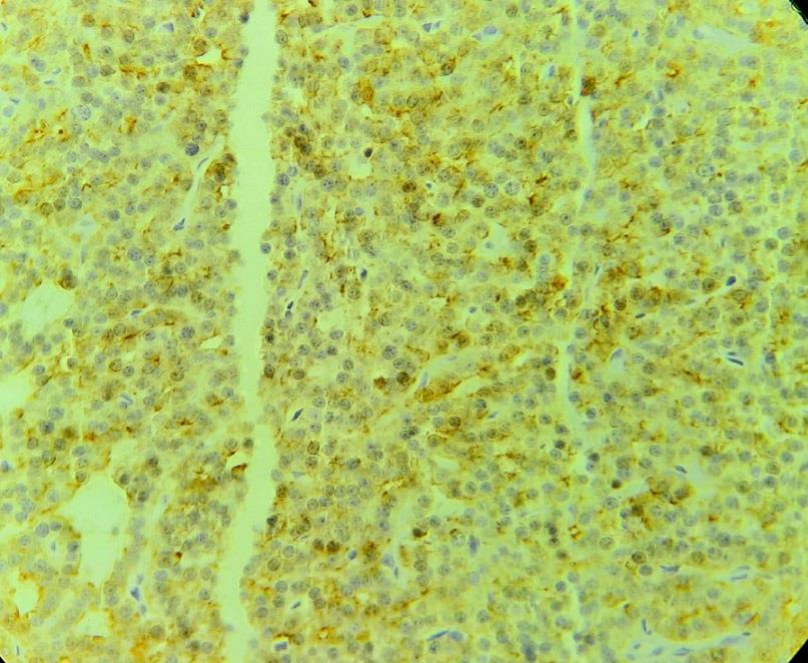
tumour cells express parathormone by immunohistochemistry

**Therapeutic intervention:** the patient underwent a total thyroidectomy with a left mediastino-recurrent curage and a homolateral laterocervical functional curage. However, a left isthmolobectomy was performed in the first instance, and an extemporaneous anatomopathological examination revealed an encapsulated oncocytic vesicular tumour. The other thyroid lobe was removed in the second stage. The left supraclavicular mass was then removed. Macroscopically, it was a hard, polylobed, encapsulated mass measuring over 4 cm in diameter, very adherent to adjacent tissues. Extemporaneous anatomopathological examination of the mass revealed no lymph node tissue, but rather an oncocytic vesicular lesion to be confirmed after kerosene embedding. The remainder of the surgical procedure included a left mediastino-recurrent curage and a homolateral latero-cervical functional curage. Definitive pathological examination of the left supra-clavicular mass was consistent with parathyroid carcinoma. The morphological appearance included fibrous tissue invasion with the presence of vascular emboli (Figure 2, Figure 3), while the immunohistochemical profile showed positive anti-PTH antibodies and negative anti-thyroglobulin antibodies (Figure 4). The remainder of the examination confirmed the diagnosis of an encapsulated oncocytic vesicular tumour in the left thyroid lobe, while the right lobe contained a hyperplastic nodule. All lymph nodes removed during curage were reactive.

**Follow-up and outcomes:** post-operative management was straightforward. Parathyroid hormone levels were normal (PTH 49 pg/ml), and the patient developed hypocalcemia to 75 mg/L, which was corrected by calcium supplementation. She was then referred to endocrinology for investigation of multiple endocrine neoplasia and initiation of hormone replacement therapy. A 99m HMDP-Tc bone scan revealed no secondary bone locations. Adjuvant external radiotherapy was not recommended, as the tumour was removed in its entirety and the surgical margins were healthy with the presence of reactive lymph nodes. No recurrence or metastases were observed after 8 months.

**Informed consent:** written informed consent was obtained from the patient for publication of this case report and accompanying images.

## Discussion

Parathyroid glands derive from the endoderm of the third and fourth pharyngeal pouches. The number and location of parathyroid glands are variable. Ectopic glands usually appear along the embryological descent path, in the carotid sheath, within the thyroid gland or thymus, in the supraclavicular fossa or upper mediastinum [4], which explains the left supraclavicular location of our parathyroid carcinoma.

Parathyroid carcinoma is a very rare malignant tumor with an estimated prevalence of 0.005% of all cancers, affecting around 0.5 to 4% of patients with hyperparathyroidism, with an extremely low incidence of around 4 to 6 cases per 10 million inhabitants per year [1,2]. The condition generally occurs between the fifth and sixth decades of life, with no sexual predominance [3]. Its etiopathogenesis remains poorly understood. Cases have been reported in the context of hereditary syndromes: multiple endocrine neoplasia type I or type IIA, or the 'hyperparathyroidism jaw tumor' syndrome associating primary hyperparathyroidism, mandibular fibro-osseous tumors, renal and/or uterine tumors [3].

The diagnosis of parathyroid carcinoma is generally delayed due to the non-specificity of clinical and biological signs, especially if it is non-metastatic. The majority of parathyroid carcinomas are secretory, and the clinical picture is often similar to that of benign parathyroid conditions, dominated by the polymorphic symptoms of severe hypercalcemia due to primary hyperparathyroidism, which explains the difficulty in differentiating them [2,5]. Certain signs, unusual in benign hyperparathyroidism, point to malignancy, such as dysphonia, dysphagia and the presence of cervical adenopathies [2,6,7]. In the case of non-secreting carcinomas, which are extremely rare, the diagnosis is even more difficult in the presence of a locally advanced cervical mass, given the absence of signs of hyperparathyroidism [3]. In our case, the patient showed no signs of hypercalcemia, but rather a left supra-clavicular mass suspicious of malignancy, which made the diagnosis very difficult.

Biologically, severe primary hyperparathyroidism is strongly suggestive of malignancy [3]. Calcemia levels average 10 times normal in parathyroid carcinoma, as opposed to 2.6 times normal in benign primary hyperparathyroidism. In our case, the patient's calcemia was borderline normal, in contrast to the above-mentioned biological criteria, confirming the non-secretory nature of this carcinoma. As for imaging, the sensitivity of cervical ultrasonography for parathyroid carcinomas varies from 50 to 90% according to several studies carried out. Parathyroid carcinoma appears as a hypoechoic lobulated lesion with irregular boundaries, containing calcifications and infiltrating surrounding tissues [3]. The absence of intratumoral vascularization, the ovoid shape and thick capsule of the lesion make malignancy unlikely [2,6]. Ultrasound examination of our patient failed to evoke the diagnosis, given the location of the mass in the left supra-clavicular fossa and the absence of clinico-biological signs of primary hyperparathyroidism. Technetium-MIBI or Tc-99m-sestamibi scintigraphy is more useful because the tracer is rapidly eliminated from the thyroid parenchyma, but persists longer in the parathyroid parenchyma. Thus, late images would correspond to a parathyroid localization [8]. It can therefore be used to localize and characterize the tumour, and to diagnose and locate lymph node metastases or distant metastases, but it does not provide information on the benign or malignant nature of the tumour.

More recently, 18F-Choline PET has been described, but its indication is controversial despite its high sensitivity [9]. In cases of strong presumption of malignancy, cervical MRI is recommended to highlight the cervical mass and study locoregional extension. Thoraco-abdomino-pelvic CT scans are used to search for distant metastases [8], which were not found in our patient. However, only a histological study can confirm the diagnosis of parathyroid carcinoma. Fine-needle aspiration should be avoided because of its low sensitivity and the risk of tumour dissemination. Macroscopically, parathyroid carcinoma appears as a solid, hard, polylobed, encapsulated, grey to whitish mass that adheres tightly to the thyroid lobe or adjacent tissues [3,7]. Microscopically, only invasiveness is a criterion of malignancy according to the new World Health Organization classification [10]. Immunohistochemistry techniques using monoclonal antibodies, PCNA and Ki67, can be used to confirm the diagnosis, but also for prognostic purposes.

Therapeutically, medical preparation can be instituted before surgery to correct severe or even malignant hypercalcemia, based on intravenous rehydration with isotonic saline, administration of Henle's loop diuretics and biphosphonates. In cases of oligoanuric renal failure, hemodialysis sessions are necessary [3,7]. The standard treatment for parathyroid carcinoma is surgical resection [2,5], comprising parathyroidectomy combined with homolateral loboisthmectomy, respecting the recurrent nerve if not invaded, and homolateral mediastino-recurrent lymph node dissection as a matter of principle. Homolateral jugulocarotid evisceration is performed in the presence of obviously metastatic adenopathies, found in around 25% of patients [2,8]. Parathyroid carcinoma is radioresistant. However, some authors recommend post-operative external radiotherapy at a dose of 40 to 70 Gy in cases of locoregional invasion, to reduce the risk of local recurrence. The efficacy of chemotherapy in the treatment of parathyroid carcinoma has not been proven. However, it can be combined with radiotherapy as a palliative treatment for inoperable or metastatic parathyroid carcinoma [3,7]. Targeted therapies such as Sorafenib can also be used to treat metastatic forms [8]. Post-operative follow-up is based on serum calcium and parathyroid hormone levels. The rate of local recurrence, even late recurrence, is estimated at between 30% and 70% according to various studies. The rate of lymph node or distant metastases, especially in the lungs or bones, is estimated at 30% of cases. Overall survival at 5 years is 85%, and at 10 years 49-77% [3,7]. In our case, extended surgical excision was performed without adjuvant radiotherapy, since the margins were healthy and the lymph nodes were reactive. Postoperative parathyroid hormone levels were normal. No recurrence or metastases were observed after 8 months.

## Conclusion

The diagnosis of parathyroid carcinoma is based on a combination of clinical, biological, radiological and, above all, histological findings. However, when the carcinoma is non-secretory, the diagnosis is even more difficult, particularly in the presence of an unusual location such as the left supra-clavicular fossa. The treatment of choice is surgery, which may be combined with external radiotherapy in advanced cases.
